# Why primatology needs local knowledge

**DOI:** 10.1007/s10329-026-01256-1

**Published:** 2026-04-27

**Authors:** The Perspectives Collective, Sam Adue, Walter Akankwasa, Jackson Asua, Gideon Atyo, Bosco Chandia, Noémie Freymond, Vicent Kizza, Harmonie Klein, Monday M’Botella, Stephen Mugisha, Geresomu Muhumuza, Mike Matthew Musiimenta, Daniel Sempebwa, Eguma Robert Yiki, Elodie Freymann, Derry Taylor

**Affiliations:** 1 Budongo Conservation Field Station, Masindi, Uganda; 2 Bugoma Primate Conservation Project, Hoima, Uganda; 3 University of Neuchatel, Neuchâtel, Switzerland; 4 Wild Minds Lab, University of St Andrews, St Andrews, UK; 5 Ecology of Animal Societies, Max Planck Institute of Animal Behavior, Konstanz, Germany; 6 Institute at Brown for Environment and Society, Brown University, Providence, USA; 7 Institute of Cognitive Science Marc Jeannerod, Bron, France

**Keywords:** Local knowledge, Traditional ecological knowledge, Epistemic diversity, Collaborative research, Field-based expertise, Knowledge production, Conservation ethics, Inclusive science, Human–primate interactions

## Abstract

Primate research has long depended on the deep ecological and behavioral knowledge of local communities living near or within primate habitats, yet this knowledge has rarely been given the same visibility as academic science. In this collaboratively formulated article we argue that including local and traditional knowledge alongside scientific studies enriches primatology epistemically, ethically, and practically. We introduce the Perspectives Collective Journal, a collaborative multimedia journal–the first of its kind–created to support the direct sharing of local and traditional insights about primates, as well as other species, in their natural habitats. By providing an accessible and flexible platform for storytelling, reflection, and documentation, including written articles, participatory films, interviews, and group discussions, Perspectives invites a plurality of voices and formats into primatological knowledge production. Here we highlight several ways in which local knowledge can contribute to primatology and its complementary relationship to mainstream science by drawing upon materials published in *Perspectives* and framing it in relation to the mainstream scientific literature on primate behavior. Overall, this paper and the Perspectives project more generally demonstrate the value of allowing diverse knowledge systems to meet on equal footing.

**Supplementary Information:**

The online version contains supplementary material available at https://doi.org/10.1007/s10329-026-01256-1.

## Introduction

For much of its recent history, primatology has been shaped by scientific institutions and publishing norms that privilege quantitative methodologies and researchers from reputed research institutes and universities. These frameworks have produced extraordinary insights—but they have also limited whose voices are heard and what forms of knowledge are recognized as legitimate. Across primate field sites, local researchers and full-time field staff possess an extraordinary depth of understanding of the animals and landscapes, owing to decades of lived experience. Such extensive, situated experience is particularly valuable for understanding primate species with high levels of behavioral flexibility, slow life-histories, and complex social and emotional lives that unfold on both short-term and long-term timescales. That is, primate lives naturally lend themselves to narrative accounts that are often difficult to fully capture scientifically because they unfold as a series of unique events which are therefore difficult to quantify. Acknowledgement of qualitative complexity within primatology has notably been a foundational principle underlying the Japanese approach to primatology, which was founded on emphasizing relational forms of knowing, recognition of the individual, and the need for long-term studies (Matsuzawa and Yamagiwa 2018).

This long-term, situated primatological experience generates insights that short-term research projects often cannot (Kappeler and Watts 2012). For example, in the Budongo Conservation Field Station (BCFS), located in Western Uganda, local staff follow two wild Eastern chimpanzee (*Pan troglodytes schweinfurthii)* groups “*every week and every day*”, with many staff members having accumulated 20–30 years or more of experience. Local field staff at BCFS are therefore able to accumulate a unique temporal and spatial depth of observation. This experiential expertise far exceeds that of most visiting researchers and foreign students who stay at the station for comparatively short-periods of time. As one BCFS *Perspectives* member noted, “*When scientists come in*,* for one month*,* two months*,* six months… time is less… but our experience makes it easy.*” Observations by local field staff capture slow ecological changes, shifting fruiting patterns, individual life histories, and behavioral development, which has been shown to take up to 15 years in some domains such as chimpanzee tool-use (Malherbe et al. 2024). As such, these elements of primate lives can be difficult (although of course not impossible) to capture scientifically, yet are crucial for understanding behavior and biology.

Yet, despite its depth and rigor, much of this local and experiential knowledge remains largely unpublished and therefore uncitable — not because it lacks validity, but because it does not easily conform to the traditional academic conventions. As another BCFS field staff co-author reflected, “*We have everything scientists need from chimps*,* but we can’t put it in the way they want to arrange it*.” This gap between what visiting scientists seek to understand and what local researchers and field staff already know is not epistemological, but structural, reflecting a much more general problem in academic publishing in the life sciences that favours quantitative over qualitative information; existing publishing systems rarely accommodate narrative, experiential, or collectively held knowledge, even when such knowledge demonstrably improves scientific understanding. Unfortunately, when local perspectives are not included in academic literature, important information is inevitably lost (Rodrigues et al., 2022). A BCFS *Perspectives* member observed: researchers who do not take local knowledge seriously “*can’t really come with a deeper understanding of what they want to know*,” and may therefore miss emerging behaviors that local observers are already well aware of.

**Fig. 1:**
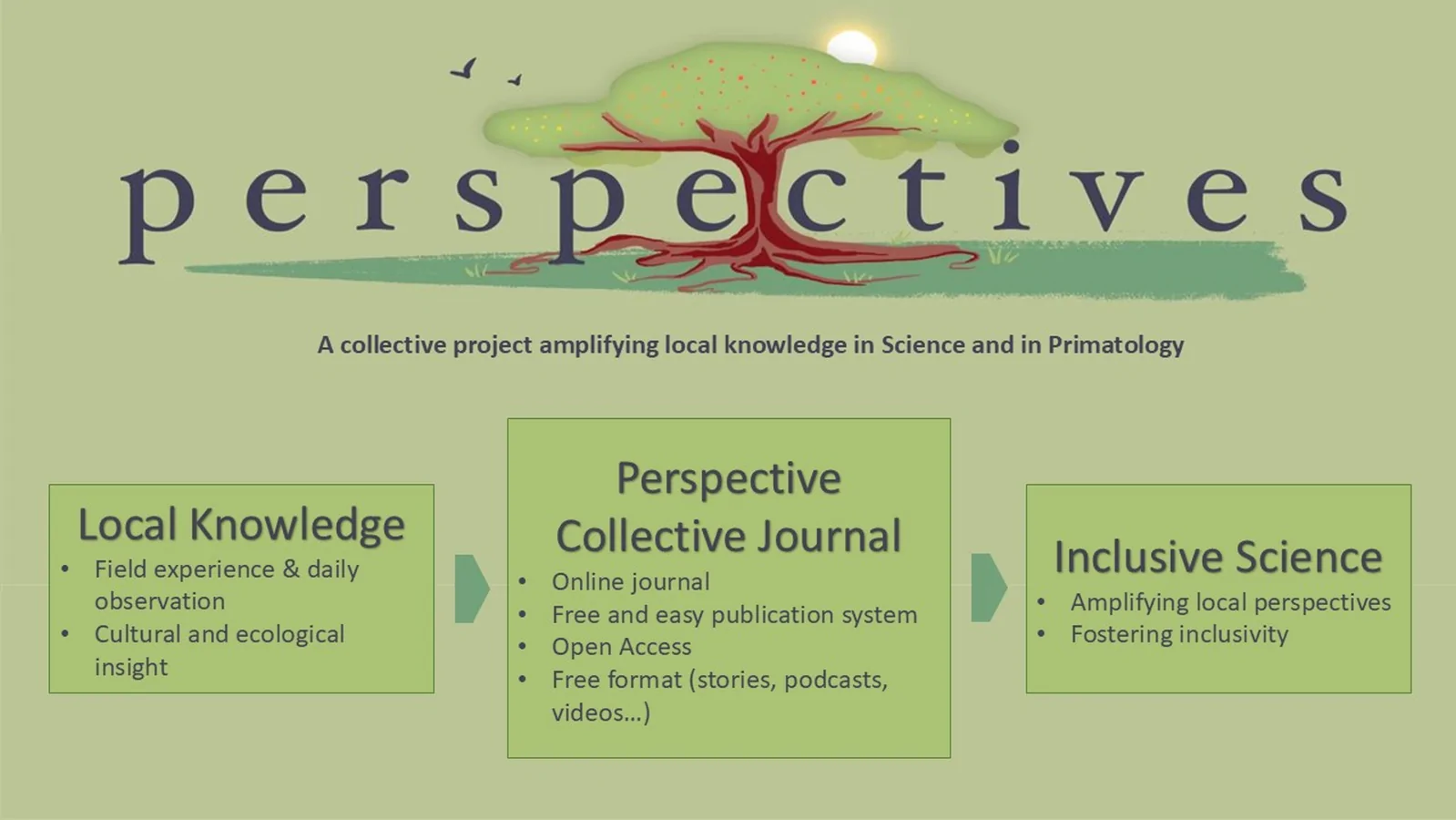
Workflow illustrating the origins, implementation, and outcomes of *Perspectives Collective Journal outputs*

In recent years, primatology has begun to move toward more inclusive models of collaboration and authorship (e.g., Miles et al. 2022; Hayes and Paparini 2023). The *Perspectives Collective* (Fig. 1), an initiative developed at BCFS, represents one such step forward. *Perspectives* provides a dedicated space for local researchers to share their experiences through writing, film, and audio, on their own terms and in their own languages. Rather than positioning local knowledge as supplementary to science, the platform treats it as an equally vital way of knowing. As several BCFS *Perspectives* members emphasized, “*We don’t just come out with what we think—we come out with what we see*,* what we have observed*.” In doing so, *Perspectives* makes visible forms of knowledge that are often assumed but rarely credited within primatological research.

Integrating local and traditional knowledge into primatology is not about replacing science, but about broadening it. Indeed, in her plenary talk at the 2018 International Primatological Society Congress in Kenya, Nairobi, Professor Shirley Strum advocated for a return of the natural history approach to primatology, drawing upon qualitative documentation of experience to fill gaps in primatology left behind by mainstream contemporary scientific approaches (Strum 2019). Scientific research and local experience each illuminate different facets of reality: science excels at systematic measurement and hypothesis testing, while local knowledge draws on sustained, practical engagement with complex ecosystems (Berkes 2017). Together, they can offer a more complete understanding of the natural world (Tengö et al. 2014). As one BCFS *Perspectives* member explained, local knowledge helps scientists move from abstract description to grounded understanding by providing “*proof of ideas*” through lived-encounters.

There is also a strong ethical imperative to recognize and amplify local perspectives. Primate research is deeply dependent on people who live and work in primate habitats, and meaningful collaboration requires more than logistical involvement and data collection. It requires respect, recognition, and intellectual partnership (Setchell et al. 2025). One BCFS *Perspectives* member emphasized how transformative it feels when their expertise is taken seriously in scientific contexts: “*It’s very important to hear that world scientists are aware of what we published…it feels amazing when others listen*.” Such recognition aligns with the United Nations Declaration on the Rights of Indigenous Peoples (2008), which affirms equitable participation in research as both an ethical obligation and a foundation for best practice.

Despite growing awareness, many structural barriers continue to shape who gets to publish and whose knowledge “counts”. Pay-walled journals, high open-access fees, English-language hegemony (Amano et al. 2023), and unfamiliar submission systems all contribute to a publishing ecosystem that remains inaccessible to many habitat-country researchers. Addressing these barriers requires new platforms that value diverse forms of expression and expertise. *The Perspectives Collective Journal* was founded as one such venue—a collaborative, multimedia platform where local researchers can share insights in the formats and languages of their choice. As contributors describe it, the journal allows the world to see knowledge that has long existed: “*We are covered by the canopy of Budongo… but through* Perspectives *the world can see our diversity of knowledge*,* which is very important for science*.” In this sense, *Perspectives* demonstrates how epistemic inclusivity can be achieved not by forcing diverse voices into existing molds, but by expanding what counts as contribution, authorship, and knowledge itself.

In this article, we explore why plural knowledge systems are vital to the future of primatology. We examine the ethical, epistemological, and practical significance of local and traditional knowledge, and we present *Perspectives Collective* as an emerging model for equitable and collaborative knowledge exchange. Our aim is not to critique primatology’s past, but to envision its evolution: a field that listens to the full range of voices that make new discoveries possible, and, in doing so, becomes both more just and more insightful.

## Ethical significance of local and traditional knowledge

Ethics in primatology have traditionally focused on the welfare of nonhuman primates, minimizing disturbance, ensuring human treatment, and promoting conservation (but see the CARE framework for a new ethical approach to studying animal behavior; Badihi et al. 2026). Yet the ethics of knowledge production within the field are equally important. Who is recognized as a “knower”, whose observations circulate in scientific literature, and who has access to authorship reflects deeper power relations within the discipline. Ethical recognition, in this context, is closely tied to the visibility and circulation of shared work. As one BCFS *Perspectives* member emphasized, *“It’s very important to feel there are others who recognise the importance of our knowledge”.* Another BCFS *Perspectives* member added: *“We feel very encouraged when we feel our knowledge is very useful not only here in Budongo but elsewhere also”*. These statements reflect more than a desire for validation, instead they highlight *Perspective* members’ strong feelings that knowledge produced through long-term local engagement deserves to travel, to be read, and to matter beyond the field site.

Ethical collaboration also requires material and practical support, particularly where structural barriers to publishing remain. As one *Perspectives* member explained, *“We struggled to find a way to publish our knowledge…but some researchers who came*,* sacrificed their time and materials to help us do it through* Perspectives.*”* Here, collaboration is described not as consultation, but as shared effort—recognizing that equitable knowledge production sometimes requires uneven contributions of time, resources, and institutional access (i.e., visitors utilizing their resources not only for their own projects, but to help others to access information and share their knowledge and experience in a way that is meaningful to them). Ultimately, through shared investment in the production of alternative knowledge forms, primatology becomes a more inclusive space, which is essential given the highly international nature of primatology combined with a strong skew regarding credit (i.e., most co-authors of primatology papers are from Europe and North America – continents with no endemic primates; Setchell and Gordon 2018).

Respect, as articulated by the co-authors of this paper is enacted through presence, responsiveness, and sustained engagement. One *Perspectives* member described what the enactment of respect looks like to them: *“When we are gathering here* [at Perspectives meetings] *to discuss and researchers come*,* we know this collaboration is nice*,* we feel respected because our knowledge is being valued”.* These reflections underscore that ethical collaboration is relational, unfolding through repeated interaction rather than one-off encounters – it is embedded into our research routines. In field primatology, research is a daily activity that takes place over large time scales (daily data collection protocols at many long-term primate field sites have been running consistently for decades) through consistent daily focal follows, providing the perfect opportunity to create the kind of routines that foster respectful and mutually gratifying collaborations.

Ethical and equitable collaboration in primatology means that all participants are treated as co-creators of knowledge. One BCFS *Perspectives* member noted, *“If you*,* as a researcher*,* can build more knowledge than others from us*,* then we are very happy with this collaboration.”* Another added *“… because we know what we know is not useless*,* it is not in vain. We know we have realised something beyond others”.* These sentiments help illustrate that while many non-local academic primatologists have taken positive steps to ensure field collaborators are equally included as co-authors on academic publications where appropriate (Hobaiter et al. 2021), there is also a critical need to recognize the importance and contribution of local knowledge in ways that are more meaningful than just nominal.

When primatology is practiced equitably, it can positively reshape how local researchers are seen—both by others and by themselves. As a *Perspectives* member explained: *“I think it helps the foreign researchers to also know that we carry a lot of knowledge on what they’re doing*,* it’s just that we can’t put it the way they always think of putting it*,* but it shows we are like one of them who are supposed to be taken as important.”* Others emphasized the confidence that comes from this recognition: *“It makes us more confident about our knowledge.”* Another member reflected specifically in regards to *Perspectives*, that it makes him *“ seen as someone who is international*,* who works with international researchers*,* not just local”.* Making local and traditional knowledge visible and valued, therefore, makes a positive contribution to the wellbeing and sense of self to those who participate in the field.

Ultimately, ethical primatology is not only about what is studied, but about how knowledge is shared and who is enabled to speak. In this collaborative context, ethics is practiced through inclusion, support, and shared authority over the research. Recognizing local and traditional knowledge, then, is not an ethical supplement to primatology, but a reconfiguration of how the field understands collaboration, expertise, and responsibility.

## Epistemological significance of local and traditional knowledge

At its core, science is a way of seeking understanding about the world around us. It depends on observation, interpretation, and the ongoing testing of ideas against experience. In this sense, scientific and local ways of knowing share a common foundation: both are grounded in sustained engagement with the natural world. What differs are the frameworks through which observations are made meaningful—the questions asked, the categories used, and the languages in which insights are expressed. On this point, one *Perspectives* member remarked: “*When I understand the scientific language*,* understanding is no problem*,* once the vocabulary has been broken down*,* the only problem then I feel is sharing the local experience in relation to the research topic*”. Therefore, acknowledging the multiplicity of epistemic traditions does not weaken scientific inquiry; rather, it provides an alternative, but equally valuable and scientifically relevant point-of-view.

For the co-authors of this paper, local knowledge is rooted in direct encounters with the forest and its many inhabitants, through extensive training, daily experience, and in some cases local ecological knowledge acquired outside of the workplace. As one *Perspectives* member articulated, *“Local knowledge is knowledge you acquire at home*,* wherever you go with your friends*,* and here at work*,* where we see the chimpanzees directly by our own selves*,* without someone directing you”.* This formulation foregrounds an epistemology based on firsthand observation rather than mediated instruction. Knowledge emerges not through predefined research protocols alone, but through repeated presence, attentiveness, and familiarity. Unlike many forms of scientific knowledge which seek to separate the observer from the observed in the name of objectivity, local knowledge is embodied. One *Perspectives* member stated “*We carry almost all our knowledge in our head”*, making this knowledge uniquely situated within the given ecological, social, cultural landscape. Given the extensive cross-population variability in the behavior of most primate species (Basava et al. 2025), situated forms of knowledge are clearly highly relevant to the field of primatology.

Local knowledge is accumulated over vast spatio-temporal scales, spanning decades not only within the forest, but in some cases, also in the larger socio-ecological system the forest is embedded in, a wider world that local researchers and field staff are often enmeshed within, providing a richer, broader perspective of animal behavior and ecology. “*We experience chimpanzees at home as well as inside the forest*” one *Perspectives* member remarked. Indeed, many local community members possess knowledge of chimpanzees and other primates owing to their tendency to leave their forest habitat to raid crops (Naughton-Treves et al. 1998), although the knowledge gained through such encounters is typically an incomplete picture of chimpanzee behaviour (Sousa et al. 2014). Routine engagement with chimpanzees at work and in the villages can allow local researchers and field staff to notice patterns and changes that visiting researchers focused on behavior in the forest may not capture, by combining knowledge gained through training and research experiences, with local experience outside of the work place. As one *Perspectives* member explained, *“The experience we acquire in the wild with chimpanzees for a long time*,* means that when scientists come in*,* you first want to follow the chimpanzees to know more about them*,* we already have the knowledge because we follow them every week and every day.”* This depth of observation produces a holistic form of empirical understanding grounded in unbroken continuity between the inside and outside of the forest, an epistemic resource that complements rather than competes with structured scientific data.

The epistemic contribution of local knowledge is often most apparent in contexts where it is questioned or dismissed by researchers. As several scholars have noted, many researchers—particularly early-career scientists—tend to privilege scientific knowledge while doubting the observations and experiential insights of local assistants, especially when these do not align with established behavioural “facts” or research agendas (Cardinal et al. 2022). Such scepticism reflects a narrow definition of knowledge that values formalized data over sustained, place-based observation. In contrast, *Perspectives* members repeatedly emphasized that their long-term engagement with local ecosystems enables them to identify patterns, contextual factors, and exceptions that are often overlooked by short-term scientific studies. In this sense, local knowledge is not merely supplementary to scientific research; it plays a critical epistemic role by questioning dominant assumptions, refining interpretations, and expanding what is recognised as valid knowledge within scientific practice.

At the same time, *Perspectives* members are clear about the boundaries of different knowledge systems. As one reflected, *“We have everything scientists need from chimps*,* but we can’t put it in the way they want to arrange it.”* This is not a deficit, but an epistemological distinction: local knowledge is often held narratively, practically, and collectively, rather than in the formalized structures favored by academic science. Recognizing this difference allows scientific inquiry to expand its interpretive repertoire without demanding epistemic conformity. While some primatological journals (e.g., Pan Africa News) embrace more informal anecdotal reports, this assumes a degree of academic literacy that some local field staff may not have training in. While the attempt at inclusivity is certainly a positive step, its efficacy is nonetheless limited. Platforms like *Perspectives* make a wider range of contributions visible and citable by allowing knowledge to be shared in forms that align with how it is produced and held. By legitimizing narrative, oral reflection, and collective discussion alongside written analysis, *Perspectives* broadens what counts as knowledge without abandoning empirical grounding.

Recognizing the epistemological significance of local and traditional knowledge does not imply a rejection of the long-term empirical foundations of primatology. Rather, it invites a broader understanding of how knowledge about primates is produced over time through the combined efforts of researchers and local collaborators. Primatological research is often built on decades of sustained observation, involving both scientists who dedicate their careers to particular field sites and local staff whose continuous presence enables data collection, interpretation, and contextual insight. Acknowledging these multiple forms of engagement shifts primatology away from privileging a single epistemic standpoint and toward a more reflexive practice that listens across differences. Such an approach does not dilute scientific rigor; it deepens it by recognising that knowledge emerges through long-term, collaborative, and situated relationships with both human and non-human actors.

## Practical significance of local and traditional knowledge

Making primatology much more inclusive is not only an ethical and epistemological goal, but it is also a profoundly practical one. When diverse voices and positionalities are represented, the discipline gains access to a wider range of experiences, insights, and problem-solving approaches. Inclusion enriches our collective understanding of primate lives and supports more effective research, conservation, and field practice. Beyond its moral and intellectual value, epistemic pluralism offers concrete benefits for research quality, interpretive accuracy, and long-term sustainability.

Local expertise is foundational to the everyday practice of primatology. Field research depends not only on methodological design, but on knowing how to move through the forest, how to follow animals safely, and how to interpret subtle environmental cues. As one co-author succinctly put it, *“I can go in the forest alone and come back alone. Someone else might go inside and get stuck there.”* This practical familiarity with terrain, routes, and risks is not supplemental to science; it is what makes field science possible. In primatology, this is particularly important, as many species, such as mandrills (*Mandrillus sphinx*), golden snub-nosed monkeys (*Rhinopithecus roxellana*), and chimpanzees have home range sizes that can range from 5km^2^ up to 200km^2^ or more (Rogers et al. 1996; Baldwin et al. 1982; Yao et al. 2022), presenting an immense challenge when it comes to tracking wild primates and safely collecting data.

Local knowledge also contributes directly to the accuracy and efficiency of scientific research. Long-term familiarity with chimpanzee behavior allows local researchers to anticipate movements, identify individuals, and contextualize observations in ways that short-term visits cannot. One *Perspectives* member explained how this experience supports visiting scientists: *“When scientists come in*,* for one month*,* two months*,* six months…time is less…but our experience makes it easy.”* This continuity of observation and personal familiarity with not only the landscape, but the personalities and habits of individual animals and their social networks, in relation to the wider socio-ecological setting, enables research projects to begin from an informed baseline rather than from trial and error. In species where group sizes can be up to several hundred individuals, as is the case in some chimpanzee groups and monkey species including mandrills and geladas (*Theropithecus gelada)*; (e.g., Martinez-iñigo et al., 2022; Abernethy et al. 2002; Snyder-Macklet et al.  2012), knowing individual animals, as well as their goals and habits is essential to successfully obtain adequate samples from relevant individuals in the desired context. The utility of this predictive ability was affirmed by one *Perspectives* member who described how their observations are repeatedly validated over time: *“They also see that what is said by the field assistant eventually comes true without any doubt.”* This statement underscores that local knowledge is not speculative or anecdotal, but grounded in repeated empirical confirmation through daily engagement with animals and landscapes, and capable of making accurate predictions in a complex environment.

Local knowledge also plays a key role in identifying emerging behaviors and ecological change. Because local researchers observe chimpanzees across seasons and years, they often notice developments before these are captured in formal datasets. As one *Perspectives* member explained, *“What we see with the chimpanzees*,* there are things they can do*,* but it takes a while for it to happen”.* Indeed, some primate behaviors are observed just a handful of times in decades of observation by a single individual (e.g., there have only been 15 cases of tool-use by a single Bonnet macaque (*Macaca radiata*) over more than 20 years of observation in Bandipur National Park, India; Sinha 2004). Another *Perspectives* member highlighted the practical consequences of academic scientists not listening to expert field staff: *“If they don’t listen to the local knowledge*,* they are going to miss out*,* like what plants they can go for*,* for example”.* This highlights how primate feeding lists may be incomplete without local knowledge, as plant species or other food resources that are rare in primate diets may be overlooked by visiting researchers (e.g. Mugisha et al., 2016). For example, in BCFS, field staff have seen chimpanzees consuming snail shells on a few occasions, a dietary item that, according to traditional beliefs, is thought to have possible medicinal properties. However, this anecdotal information has not been “officially” recorded in the site’s long-term data and is therefore absent from any systematic analysis. These insights are essential for adapting research questions, conservation strategies, and management decisions.

From a conservation perspective, the practical value of local and traditional knowledge is equally clear. *Perspectives* members emphasized their awareness of environmental variability and ecological relationships, including changes in fruiting patterns and their effects on chimpanzee foraging. As one member noted, *“We have knowledge of things people don’t expect can happen – like changes in the fruiting patterns and how this changes the chimpanzee foraging behavior.”* Such observations are critical for conservation planning, particularly in rapidly changing ecosystems where historical baselines may no longer apply. Scientific studies have confirmed, using long-term data, that many of the fruit trees chimpanzees feed on show high levels of inter-individual differences in fruit production timing (Janmaat et al. 2016), which may indicate wider ecological shifts that are otherwise difficult to detect in the short term.

In this sense, the wider inclusion of local and traditional knowledge within primatology can serve as one of the field’s most effective tools. Inclusion can strengthen research design, improve field safety, enhance conservation outcomes, and ground scientific inquiry in lived reality. A primatology that listens to and collaborates with local knowledge holders is not only more equitable—it is more adaptive, more reliable, and better equipped to respond to the complexities of shared human–primate landscapes.

## The creation of The Perspectives Collective Journal: a way forward

Local and traditional knowledge has long been essential to primatology. Pioneering field researchers such as John Mitani and the late Christophe Boesch emphasized the importance of observing animals without rigid theoretical boundaries or preconceptions in order to truly understand their lives (Mitani, 2024; Crockford et al. 2024). While several studies have drawn on interviews with local communities to document their understandings of primates (e.g., Filho et al., 2018; Waters et al. 2022), and primatological field stations such as BCFS keep an ‘events book’, wherein rare and interesting events are qualitatively recorded, there has been, until now, no formal platform dedicated to recording and sharing local knowledge directly within the field of primatology. Because local knowledge often takes forms that do not align with conventional scientific publishing formats, translating it into standard academic language, whether through quantitative or qualitative methods, risks losing its depth, context, and distinctive perspective.

To address this gap, we have developed a new platform for local and traditional knowledge in primatology: *The Perspectives Collective Journal* (www.perspectivescollectivejournal.com*).Perspectives* is a free-format, open-access, community-run journal designed to share insights from those with lived, long-term experience of the primates and landscapes in which research takes place. Contributions to date have included single-author written reflections (Muhumuza 2023a, b), group-written papers (Perspectives Collective 2023a, b), participatory short films (Yikkii, 2023; Mugisha, 2025), academic posters (presented at International Primatological Society Congress, Madagascar, 2025), structured interviews (Muhumuza 2023a, b), and audio-recorded group discussions (Perspectives Collective 2023a, b). Moreover, all contributions are archived using Zenodo, giving each contribution a DOI and reference, making local knowledge directly citable in the mainstream of academia.

Since the project began at a long-term chimpanzee research site (Reynolds et al., 2005), many early contributions focus on chimpanzee behavior. However, *Perspectives* is open to all primate field sites as well as other species, accepts submissions in any language, and uses a simple, accessible submission process via WhatsApp or email. The most recently published issue expanded beyond chimpanzee behavior to on focus on ecological storytelling by local researchers and rangers in Ranomafana National Park, Madagascar, and we strongly encourage further participation from other research sites to help build this growing, community-driven dimension of primatology.

Wild chimpanzee behavior remains an extraordinarily complex topic to study. Behavioral patterns are shaped by interacting ecological, social, and cultural factors across lifespans that may span half a century (Boesch and Boesch-Achermann 2000). Consequently, some behaviors are difficult to observe or record systematically, leading to sparse datasets for many aspects of chimpanzee life. The outputs from *Perspectives* offer complementary insights—highlighting behaviors that are challenging to study through short-term or strictly quantitative field methods, either because they are rare or because they require deep, situated familiarity with the forest and its inhabitants.

For example, chimpanzee births are seldom witnessed, as females typically give birth in isolation. As a result, observations of live births are rare in the scientific record (Kiwede 2000; Soldati et al. 2023). In a recent *Perspectives* article, drawing on over four decades of collective field experience, the BCFS *Perspectives* group (2023) documented several such events in detail, providing new information on an aspect of chimpanzee life that had previously been scarcely described. Similarly, Adue (2024) reports a newly observed behavior—chimpanzees checking jackfruit ripeness during crop raiding in local villages using a technique used by local people. Adue’s (2024) observations concur with a recent scientific study, showing that wild chimpanzees do indeed prefer to consume jackfruits at a specific stage of ripeness (Bowland et al. 2025). This reaffirms that local knowledge can contribute cutting-edge insights to the field and underscores the importance of co-design of research drawing upon different systems of knowledge to break new ground in primatology. Based on long-term observation and local insight, Adue (2024) suggests that this behavior may have been learned by watching humans use similar techniques, offering an intriguing hypothesis for inter-species cultural transmission and illustrating the potential for local knowledge to generate new lines of research.

Local and traditional knowledge can also illuminate phenomena that conventional field methods struggle to capture in full detail because of their rarity and complexity. For instance, inter-group encounters among chimpanzees are rare events but are particularly rich in behavioral information. Because local field staff work daily in the forest over many years, and because several individuals may be present across multiple such events, they accumulate a breadth of experience that allows for detailed collective discussions of these encounters. In a group discussion recorded by the BCFS *Perspectives* group (2023), experienced fieldworkers provided rich accounts based on shared experiences during inter-group conflicts, of group formations and tactics for both coordination and deception observed during these conflicts—offering insights that, while rarely represented in formal datasets, can guide new hypotheses and field approaches.

Some contributions also explore topics that lie outside the usual scope of quantitative research, yet nonetheless deepen our understanding of chimpanzee societies. In one article Muhumuza (2023a, b), a veteran field researcher with over 34 years of experience at BCFS, reconstructs the life history of a single chimpanzee through decades of personal observation. Such narrative accounts capture dimensions of behavior and social change that elude purely metric approaches. For example, the lived context behind dominance transitions that are often represented only as changes in Elo ratings which assign numerical scores to individuals based on the outcomes of agonistic interactions (Albers & Vries, 2001).

All scientific work exists within a broader social and cultural context, though this is rarely visible in formal publications. Academic writing tends to present an ‘objective’ point of view, while its personal, cultural, and environmental embeddedness are usually left unspoken (Riley 2019). This is particularly limiting in wildlife sciences, where animal habitats are embedded in a wider socio-ecological system that matters for ecological dynamics and, in turn, the behavior and biology of the animals we study (Rodrigues et al., 2022). *Perspectives* aims instead to make these contexts visible. Two recent short films, by Yikkii (2023) and Mugisha (2025), offer powerful examples. Both authors take viewers from their homes into the forest and back again, depicting not only their daily work with chimpanzees, but also their wider lives in and around the Budongo forest. Such examples of narrative media highlight how art and storytelling can extend primatology’s methodological repertoire and deepen our understanding of what it means to study primates in shared landscapes.

Taken together, the *Perspectives Collective Journal* demonstrates that local and traditional knowledge provides not only cultural and ethical enrichment but also substantial practical and epistemological value. These perspectives expand what primatology can observe and interpret, revealing dimensions of behavior, context, and meaning that complement scientific data. By providing a space for such contributions, *Perspectives* aims to make primatology both more inclusive and more comprehensive, uniting diverse ways of knowing to better understand and conserve our closest living relatives.

## Conclusion

To conclude, we strongly believe that academic primatology can greatly benefit from an epistemic shift in our consideration of local perspectives. At present, some of the structures that shape scientific research make it difficult for certain voices, especially those with deep local experience, to be heard. Building a more socially and epistemologically inclusive field, therefore, has both ethical and practical value: it makes our research more reflective of the contexts in which it operates (e.g., Rodrigues et al., 2022), and more capable of capturing the full richness of primate lives. Through initiatives such as the *Perspectives Collective Journal*, we aim to help expand the forms of knowledge that primatology acknowledges and preserves. By providing a platform where local and traditional knowledge can stand alongside conventional scientific research, we hope to encourage new forms of collaboration, insight, and creativity. In doing so, primatology can continue to grow into a discipline that listens as carefully as it observes—one that studies coexistence by practicing it.

## Supplementary Information

Below is the link to the electronic supplementary material.

Supplementary Material 1

## Glossary

Perspectives: The perspectives project broadly, including the journal and all of its members.
Perspectives Collective Journal: The journal created and run by Perspectives members, dedicated to sharing and documenting local insights into primates and other species.
