# Supplementary material for: Why primatology needs local knowledge
Source: Primates. 2026 Apr 27;67(5):473–82. doi: 10.1007/s10329-026-01256-1 (PMC13672654; doi:10.1007/s10329-026-01256-1)
Supplement: Supplementary file 1 [file 10329_2026_1256_MOESM1_ESM.docx]

1. What is local knowledge?
2. How does your experience help scientists understand chimpanzees?
3. What do scientists miss when they don’t listen to local knowledge?
4. For you, what is the difference between your knowledge of the forest and the chimpanzee, compared to the knowledge of a foreign researcher?
5. Why is it important to recognise local knowledge?
6. What do you want people to learn from reading *Perspectives*?
7. What does good collaboration look like to you?
8. What makes you feel respected in a research collaboration?
9. Do you always understand well the research questions of foreign students?
10. How important is it for local people to help decide what is studied in the forest?
11. Do you generally feel recognized as chimpanzee expert by foreign researcher working with you?
12. How do you think *Perspectives* changes the way local researchers are seen?
